# Recent progress on underwater soft robots: adhesion, grabbing, actuating, and sensing

**DOI:** 10.3389/fbioe.2023.1196922

**Published:** 2023-08-08

**Authors:** Yeming Zhang, Demin Kong, Yan Shi, Maolin Cai, Qihui Yu, Shuping Li, Kai Wang, Chuangchuang Liu

**Affiliations:** ^1^ School of Mechanical and Power Engineering, Henan Polytechnic University, Jiaozuo, China; ^2^ School of Automation Science and Electrical Engineering, Beihang University, Beijing, China; ^3^ School of Mechanical Engineering, Inner Mongolia University of Science and Technology, Baotou, China

**Keywords:** underwater robot, soft robotics, underwater manipulation, soft manipulator, biomimetics, bioinspired robotics

## Abstract

The research on biomimetic robots, especially soft robots with flexible materials as the main structure, is constantly being explored. It integrates multi-disciplinary content, such as bionics, material science, mechatronics engineering, and control theory, and belongs to the cross-disciplinary field related to mechanical bionics and biological manufacturing. With the continuous development of various related disciplines, this area has become a hot research field. Particularly with the development of practical technologies such as 3D printing technology, shape memory alloy, piezoelectric materials, and hydrogels at the present stage, the functions and forms of soft robots are constantly being further developed, and a variety of new soft robots keep emerging. Soft robots, combined with their own materials or structural characteristics of large deformation, have almost unlimited degrees of freedom (DoF) compared with rigid robots, which also provide a more reliable structural basis for soft robots to adapt to the natural environment. Therefore, soft robots will have extremely strong adaptability in some special conditions. As a type of robot made of flexible materials, the changeable pose structure of soft robots is especially suitable for the large application environment of the ocean. Soft robots working underwater can better mimic the movement characteristics of marine life in the hope of achieving more complex underwater tasks. The main focus of this paper is to classify different types of underwater organisms according to their common motion modes, focusing on the achievements of some bionic mechanisms in different functional fields that have imitated various motion modes underwater in recent years (e.g., the underwater sucking glove, the underwater Gripper, and the self-powered soft robot). The development of various task types (e.g., grasping, adhesive, driving or swimming, and sensing functions) and mechanism realization forms of the underwater soft robot are described based on this article.

## 1 Introduction

In recent years, the development of underwater soft robots can be roughly classified according to different tasks, including soft adhesion, soft gripper, soft actuator, and soft sensor:

(1) Soft adhesion is generally an adhesion cavity composed of some viscous flexible materials, special structures (Chen et al., 2020), or both, which can be roughly divided into adhesion caused by material viscosity and vacuum adhesion formed by structural characteristics. Nowadays, soft adhesion development generally relies on morphological studies of organisms known to be capable of adhesive (e.g., the octopus-type adhesive that looks like a cup, the remora-type adhesive that has stiff spines and an outer edge of attachment, and the clingfish-type adhesive that has outer edge adhesion sucker). The basic idea of soft adhesion is to imitate existing organisms (reconstruct in terms of structure and operation mechanism effects) with known adhesion capacity to achieve certain adhesion in a specific environment (Kang et al., 2021).
(2) Soft gripper, as the main purpose of grasping, is generally intended to better collect resources. In particular, in the underwater environment, when the object we need to grasp is too fragile (Billard and Kragic, 2019), how to grasp it effectively has become a research hot spot in the application of flexible grippers. In this context, the structure of a flexible gripper can include a wrist, finger, and finger contact surface. With the continuous exploration of material technology, the gripper is also attached with flexible materials (e.g., rubber and deformation metal), from the original rigid gripper with a pure rigid contact surface to the flexible fluid-driven gripper, which is attached with the high elastic nano-finger surface. In addition, to better adapt to different application situations, some of the soft grippers studied at the present stage also retain some rigid structures, namely, rigid and soft grippers, to make up for the lack of grasping force of soft fingers and the flexibility of the inflexible structure, thereby broadening the application range of the whole soft gripper mechanism (Negrello et al., 2020).
(3) Soft actuator: The ways in which underwater life swims are varied and inspiring. Researching soft actuators allows us not only to understand how underwater life works, but also to better explore the underwater world from a different perspective. At present, there is a huge variety of soft driving structures under water, and the main driving categories are locomotion drive, jet drive, and underwater crawling or floating drive. For example, the locomotion drive of fish is subdivided into median and/or paired fin (MPF) locomotion (typically represented by manta rays) and body and/or caudal fin (BCF) locomotion (typically represented by most fish and water snakes) (Raj and Thakur, 2016). The jet drive is typically represented by jellyfish and octopuses, and the underwater crawling or floating drive is typically represented by creatures such as starfish and sea spiders.
(4) Soft sensor: Combining existing piezoelectric technology, hydrogel synthesis technology, and resistance and volume characteristics of special materials, skin-like soft sensors have also been well-developed in underwater environments in recent years. In terms of underwater soft sensing, two main sensors have been widely studied in recent years (Wang Qian et al., 2021). One is the flow sensor that mimics the lateral line of fish, whose main function is to measure the edge velocity of the sensor (i.e., to simulate the flow velocity perception of underwater fish). Another is an underwater contact sensor based on hydrogel materials, mainly imitating the pressure perception of skin touch (Giordano et al., 2021).

**FIGURE 1:**
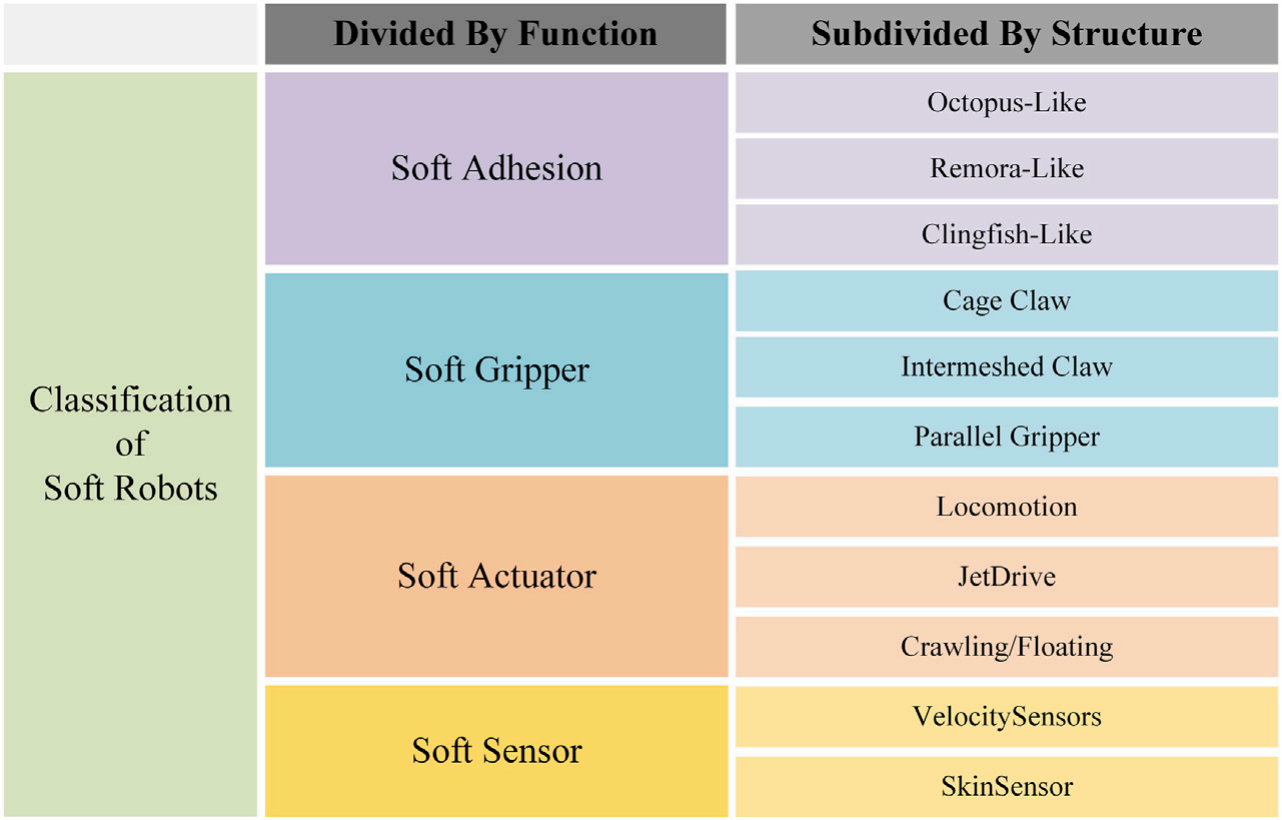
Main structure of this study.

In this review, we mainly discuss the common motion patterns of many underwater organisms and focus on recent developments in this field according to the classification of the same motion pattern. In the field of soft adhesion, soft suckers with adhesion capacity, such as octopus-like, remora-like, and clingfish-like adhesion, are highlighted. In the field of soft grippers, this paper focuses on the tentacles of the octopus and the soft grasping claws of mammals. In the field of soft actuators, the oscillating drive of fish, the jet drive of jellyfish, and the soft drive of the underwater crawling or floating motion of starfish and sea spiders are introduced. In the field of soft sensors, the development of flow or pressure sensors that mimic the lateral line of fish and the tactile sensation of skin is described. Finally, the challenges and prospects of soft robots in future underwater applications are summarized. The main structure is shown in Figure 1.

## 2 Soft adhesion

In terms of soft adhesion, inspired by octopus, remora, starfish, and other organisms, scholars have conducted a study focusing on the morphology and kinematics of these organisms and then developed it into a major direction of flexible robots at present. In the field of adhesion, this paper divides adhesion into active and passive. The main way to distinguish the two is that active adhesion needs external energy for adhesion.

### 2.1 Octopus-like

By studying the structure of octopus suckers (Tramacere et al., 2014a; Tramacere et al., 2013; Tramacere et al., 2012), the understanding of the mechanism of octopus suckers has been improved to some extent. Therefore, based on the development of flexible materials, research on some cup structure (octopus structure) suckers (Tramacere et al., 2014b; Tramacere et al., 2015) is further promoted. Certain research achievements have been made in recent years, whether it is dry-type, wet-type (Qiao et al., 2018; Wang and Hensel, 2021; Wang Yue et al., 2020), or dry and wet dual-purpose (Wang Yue et al., 2019) suckers. Under the development of passive adhesion, some progress has also been made in a series of active adhesion.

**FIGURE 2:**
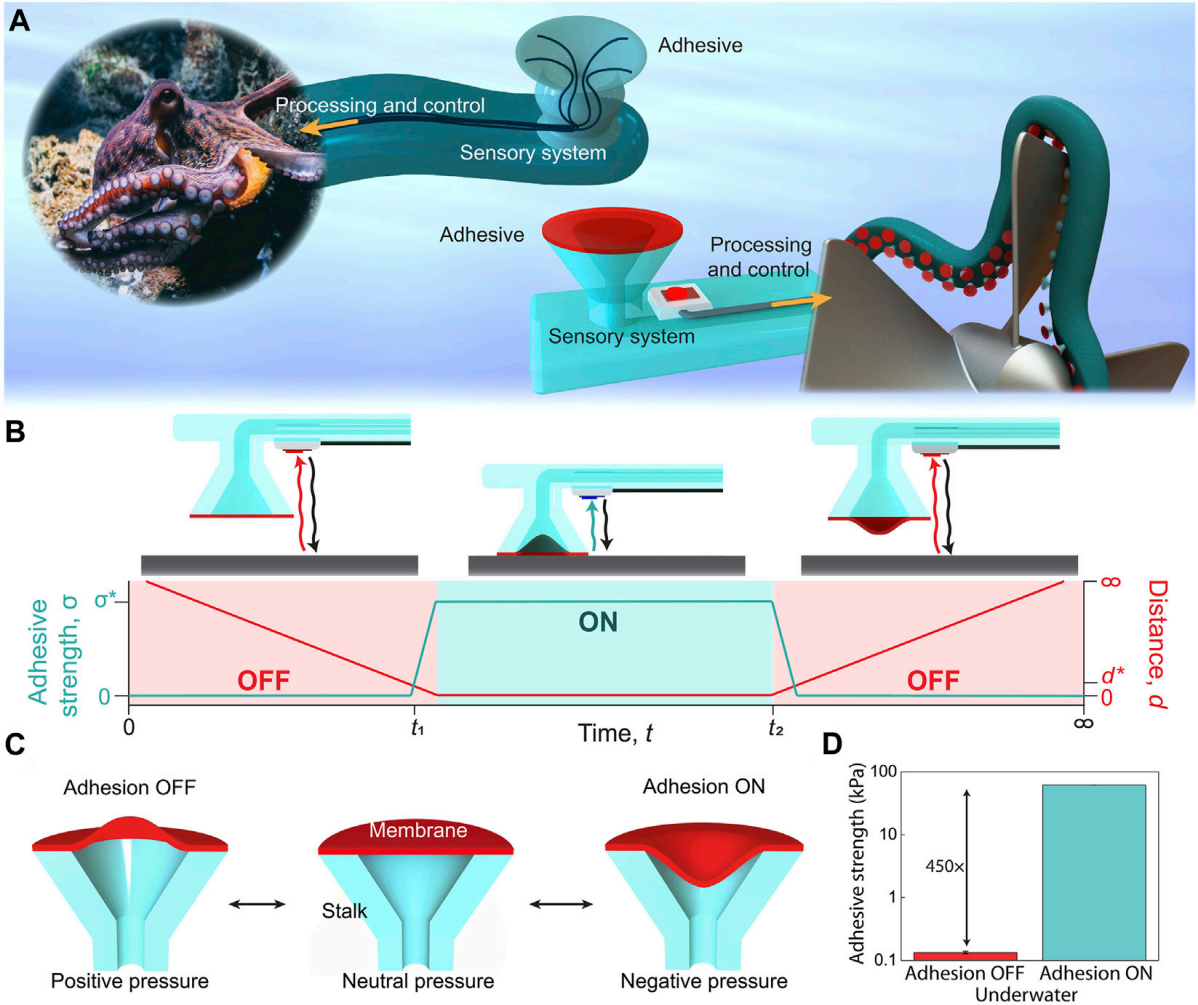
Octopus-inspired switchable and sensorized underwater adhesive. **(A)** Illustration of the octopus adhesive system and sensorized and octopus-inspired adhesive system, showing the adhesive and sensory system integrated with processing and control to sense objects and switch adhesion. **(B)** Schematic showing a synthetic adhesive with an integrated micro-LIDAR optical sensor, in which the adhesion goes from an OFF state to an ON state with an adhesive strength σ* once the sensor is triggered at a distance d*. **(C)** Schematics showing the different states of the pneumatically adhesive membrane, which controls the adhesion from the OFF to ON states. **(D)** Underwater adhesion results from an octopus-inspired adhesive, showing an adhesion switching ratio of 450× from the ON to OFF states. Error bars represent the SD for *n* = 3 [reprinted with permission from Frey et al. (2022)].

Frey et al. (2022) were inspired by octopus suckers and integrated embedded sensors, processors, controllers, and other components to develop a controllable octopus sucker that can freely control surface adhesion. This sucker can be well-attached to some irregular surfaces and can perform certain intelligent control. As shown in Figure 2, when the sensor is close to the specified distance, the suction cup begins to perform vacuum adhesion. When the adhesion surface needs to be removed, the suction cup will be inflated to remove the adhesion.

Swift et al. (2020) introduced an active polydimethylsiloxane membrane, which uses pneumatic control and tunable rigidity material to study the adhesion force and used pneumatic positive and negative pressure to cooperatively control the contact stiffness and geometry to perform adhesion and release. The active polydimethylsiloxane film can be extremely extended, and the adhesion and release time is approximately 0.1 s, which is very suitable for applications requiring rapid adhesion and release.

Wang et al. (2021b) introduced a new magnetically driven and energy-saving intelligent sucker based on the elastic energy storage mechanism of an octopus sucker. This sucker has the characteristics of rapid adjustment of adhesion strength and high-speed switching of adhesion state. The main adhesion characteristic of these structures is to manipulate the magnetic particles to imitate the movement of the acetabular roof of octopus suckers.

### 2.2 Remora-like

Based on the morphology of the remora research (Gamel et al., 2019; Beckert et al., 2015; Flammang and Kenaley, 2017), researchers found that it differs from the mimic fish sucker. The biggest characteristic of this type of adhesion is the presence of lamella and spinule support to achieve adhesion. In addition, the different lamella and spinule geometry makes the shear adhesion significantly different.

**FIGURE 3:**
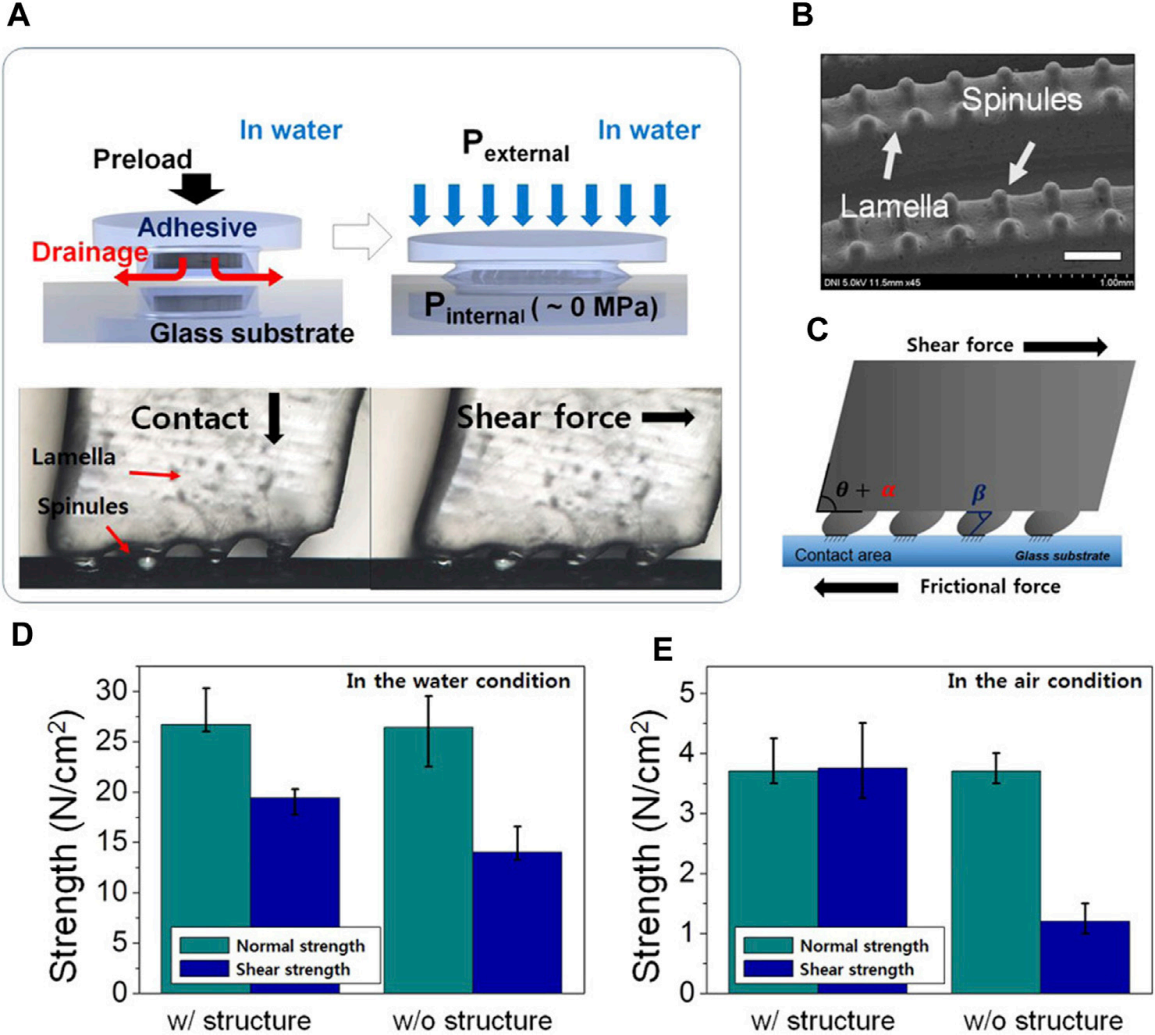
**(A)** Mechanism of adhesive force generation of the RIA and microscopic image of lamella and spinule on the glass substrate. **(B)** SEM image of the adhesive. Scale bar is 500 μm. **(C)** Deformation of the RIA. **(D)** Performance of the adhesive depending on lamella and spinule underwater conditions. **(E)** Performance of the adhesive depending on lamella and spinule under air conditions. w/ and w/o indicate the existence or nonexistence of an internal structure for lamella and spinules, respectively [reprinted with permission from Li et al. (2022).

In passive adhesion, Lee et al. (2019) made polymer-based adhesives inspired by remora suckers and conducted a series of experiments. As shown in Figure 3, the tensile and shear strengths of the adhesive model in water were 266.8 and 194.2 kPa, respectively. The adhesive maintained excellent adhesion and friction properties after multiple tests.

In active adhesion, several researchers (Li et al., 2022; Wang et al., 2017a; Wang et al., 2019b; Wang Siqi et al., 2020; Wang Siqi et al., 2022) proposed an active biomimetic sucker made of multi-material composite based on morphology and kinematics of remora, using laser processing and 3D printing technologies. This type of sucker is made of composite material and can produce higher strength than the weight of the disc. The whole mechanism is similar to the disc of live remora. Studies have shown that suckers combined with hard spines and soft materials can significantly increase the friction force on the disks, which have been tested on the substrates with different roughness.

### 2.3 Clingfish-like

Earlier studies have been conducted on the adhesion mechanism of clingfish (Green and Louise Barber, 1988), which revealed the microscopic structure and mechanism of adhesion of clingfish from a biological perspective. At the present stage, the focus is more on the study of the adhesion capacity of clingfish, and the evaluation criteria are generally the influence of dirt and surface roughness on the adhesion of clingfish (Ditsche et al., 2014; Wainwright et al., 2013). Based on these studies, scholars (e.g., Ditsche and Adam, 2019; Sandoval et al., 2019) further transformed their understanding of clingfish into artificial bionic suckers and conducted a series of tests. The prototype sucker was developed by Ditsche Petra et al., which could stick to the rock and rough surface, and the strength of the sucker was up to 70 kPa on a rough surface with a particle size of 270 μm.

### 2.4 Other mimics

In addition, except for some widely known bionic artificial suckers, researchers studied other organisms (e.g., starfish). Sandoval et al. (2022) proposed a directed adhesion sucker. Based on the directed adhesion characteristics of specific materials, they developed a type of sucker that is easy to absorb when loading in one direction and release when loading in the other direction. The characteristic of the sucker is that it can achieve obvious adhesion and detachment without other active control (Ishida et al., 2022). In other words, the sucker produces a strong adhesion capacity at a certain angle and can easily detach when the load is applied in the other direction.

### 2.5 Challenges and limitations

The main task of adhesion is to solve the problem of how to carry out the connection operation between two objects in various complex wet environments. Furthermore, the evaluation of the strength and reliability of the connection is an area of challenge. This type of surface adhesion can be applied to the docking of the underwater autonomous vehicle, the launching of the underwater autonomous vehicle, and the joint movement of master and slave robots. In conclusion, as shown in Table 1, the limitations and challenges can be summarized as follows.

**TABLE 1:** Comparison between different structures of adhesion.

Structure	Adhesion type	Adhesion object	Adhesion capacity	Material	Feature	Advantage/limitation	Reference
Octopus-like	Active	Acrylic substrate	70 kPa (max)	Silicone elastomer	Enabled by adhesives that switch adhesion state quickly with the ability to be reused over multiple cycles	Real-time object detection coupled with rapidly switchable adhesion. The control mode is too complex	Frey et al. (2022)
Octopus-like	Active	Skittles candy, tape, LEGO brick, and electrical wire	87 kPa (max)	Organosilicon material	Shrinks uniformly due to the near zero Poisson’s ratio, allowing it to preserve intimate contact during activation	Create rapid, switchable adhesion on porous, soft, and rigid objects. Need more sensors	Swift et al. (2020)
Octopus-like	Active	Frosted glass	88 kPa (balanced)	Organosilicon material	Enabled by the ability to control deformation through the stalk geometry for toughness, while being able to actively control the membrane geometry for strength and rapid release	The conversion of strong and weak adhesion can be completed in hundreds of milliseconds, limited by the preloaded magnetic field	Wang et al. (2021b)
Remora-like	Passive	Silicon molds and borosilicate glass substrates	50 kPa (max)	3D-printing materials and silicone	The disc responds to surface roughness in different manners depending on the present number of lamellae	Strong suction seal. Suction is not resistant to shear	Gamel et al. (2019); Beckert et al. (2015); Flammang and Kenaley (2017)
Remora-like	Passive	Glass surface	266.8 kPa (tensile strengths)	Silicone-based thermosetting material	The lamella/spinule hierarchical structures increase the frictional force for the RIA on the substrate	Maintained stably in the smooth surface and rough surface. The performance in air was significantly lower than that of the RIAs in water	Lee et al. (2019)
			194.2 kPa (shear strengths)				
Remora-like	Active	Acrylic substrate	15 kPa (in air)	3D-printed with different materials	Has separate lamellar compartments for redundant sealing	Perform rapid attachment and detachment on challenging surfaces both in air and under water. The adhesion force decreases significantly with the increase in surface roughness	Li et al. (2022)
			50 kPa (in water)				
Remora-like	Active	Smooth and rough surface	163.7 ± 3.8 N (on real shark skin)	Multimaterial 3D-printed	Spinule-covered, multimaterial lamellae that overlap with undercuts and overhangs	The frictional force can be significantly enhanced on both smooth and rough surfaces	Wang et al. (2017a)
			117.8 ± 0.7 N (on synthetic rough surfaces)			Unable to precisely match the mechanical properties of the disc prototype	
Remora-like	Active	Smooth and rough substrates	42.0 ± 1.8–311.8 ± 0.95 N	Multi-material 3D printing and silicone rubber	The soft fleshy lip around the disc edge, rows of lamellae along the disc length, and the bony spinules on each lamella	The internal pressure of the disc can be adjusted according to the pressure of the underwater environment. Preloading force is required during adhesion	Wang et al. (2019b); Wang et al. (2020b); Wang et al. (2022a)
Clingfish-like	Passive	Substrates of seven different surface roughnesses	60–70 kPa	Multimaterial 3D-printed	The soft-elastic disc rim, with its hierarchical structures, could adapt to the surface structure of the substrate and increase friction with the substrate	Could attach reversibly but stably to wet and rough surfaces. Suction in water is limited by cavitation	Ditsche and Adam (2019)
Clingfish-like	Passive	Irregular shapes	14.3 ± 1.4 kPa	Silicone elastomer and 3D-printed mold	The radial body geometry was capable of better-distributing stress across the circumference of the body, allowing for improved adhesion	Grip rough and irregular surfaces without necessitating sustained actuation. It is complicated to make micropillars	Sandoval et al. (2019)
Sea star-like	Passive	Smooth acrylic surface	>5 kPa	Platinum-cured silicone	Providing adhesion when loaded in one direction and releasing easily when loaded in the opposite direction	Body asymmetry and material stiffness could produce directional anisotropic adhesion. Preloads are required to produce different body symmetries	Sandoval et al. (2022)
Sea star-like	Active	Acrylic surface and flat surfaces of varying roughnesses	2.5 N	Two different silicone elastomers	Tube feet convert fluid motion into linear actuation using soft tubes that are radially constrained	Created similar performance to the use of passive suction discs without requiring high preload forces. The processing structure is difficult	Ishida et al. (2022)
Gecko-like	Passive	Glass slide	245 kPa (avg.)	Curable elastomer, organosilicon material, and TPE	The more hydrophilic a material is, the larger the reduction in adhesion performance underwater	Could controlled adhesion strength through altering surface wettability or surface tension of the surrounding fluid. Prone to internal cavitation	Soltannia and Sameoto (2014)

The main characteristics of the octopus-like suction cup are the wide range of applications (Eroğlu and Ebru Devrim, 2023) and the adjustable pressure. However, the resistance to shear stress needs to be further improved.

The remora-like sucker has added bone spurs compared to the octopus sucker. Therefore, it has better resistance to tangential force in a single direction. However, its structure is more complex, its overall structure is non-homogeneous, and the manufacturing cost is relatively high.

The clingfish-like sucker has a larger adsorption edge than the octopus-like sucker, which enables the sucker to obtain a certain good adsorption capacity with a larger surface roughness.

Furthermore, other mimic structures can make a good effort in the adhesive area (Soltannia and Sameoto, 2014), such as gecko-inspired (Soltannia and Sameoto, 2014) and sea star-inspired.

## 3 Soft gripper

As a very popular application direction of flexible mechanisms, the development of soft grippers underwater is also very promising. Particularly in grasping some vulnerable marine specimens, the soft gripper can better play its own advantages of flexible materials. In addition, the flexible gripper can be combined with the existing rigid gripper to further improve the grabbing range of targets. At the present stage, mainstream claw design inspiration comes from the construction of imitations of the human hand and other structures, such as the construction of octopus tentacles. The movement of the gripper is generally performed using the deformation of shape memory alloy (SMA) and/or soft cavity to grasp the target object. The general structure of these claws includes three parts: wrist, finger, and grasping surface. According to the different swing positions of the fingers, the gripper can be subdivided into parallel gripper, intermeshed claw, and cage claw.

The classification standard of the gripper is arranged according to the analysis of Mazzeo et al. (2022), in which the parallel gripper refers to the gripper whose fingers keep parallel when opening and closing. The intermeshed claw is a claw with three or more fingers with the same end, rotating around the end, and the edges of the fingers have certain contact. The cage claw refers to the combination of multiple fingers to envelope a certain area.

### 3.1 Basic part introduction

With regard to wrist research, Gong et al. (2018a) designed a cylindrical soft arm structure with a corrugated texture and drove it pneumatically. They conducted underwater research under laboratory conditions and quantitatively analyzed the open-loop kinematic model performance of this model. The position error caused by the open-loop model control was tested without visual and sensor feedback.

In addition, Kurumaya et al. (2018) designed a cylindrical, flexible wrist joint structure that uses a pneumatic drive under normal atmospheric conditions but uses a hydraulic drive for functional testing under a high static pressure environment (equivalent to at least 2,300 m water depth pressure).

**FIGURE 4:**
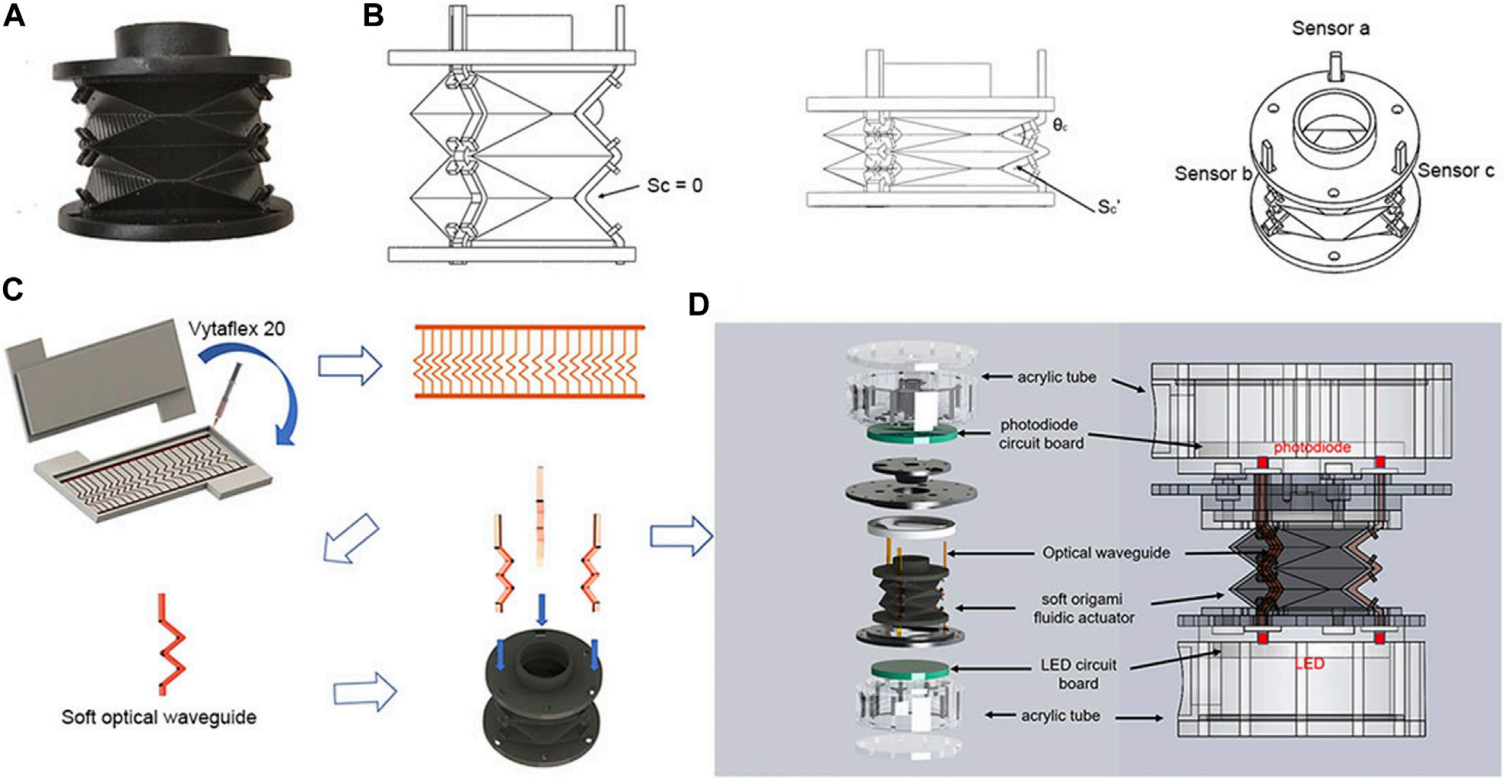
Fabrication process of the SOSA actuator. **(A)** Prototype of the soft origami optical sensing actuator. **(B)** Schematic drawing of SOSA. Sc is the contact area between the upper and lower surfaces of the optical waveguide. **(C)** Soft optical waveguide fabrication process. **(D)** SOSA exploded view and assembly [reprinted with permission from Shen et al. (2021)].

In addition to the cylindrical wrist, Shen et al. (2021), inspired by origami technology, developed a wrist with driving and sensing functions, which is controlled by fluid, as shown in Figure 4. The origami process throughout the wrist enables excellent high-load output and robust and accurate sensing performance without the need for external cladding of traditional sensors, greatly simplifies the fabrication process, and enables the wrist to be applied to complex interactive tasks in harsh environments.

Sun et al. (2020) studied the bending degree of soft, curved fingers driven by the typical bellows tube under different pressure conditions and concluded that the external water pressure could make the actuator bend more under different external pressures (1 atm∼15 MPa). In other words, the increase in the bending angle is positively correlated with the environmental pressure.

For the study on claw surface, Jiang et al. (2021) proposed seven finger surfaces designed by observing the claw shape of Boston lobster and made them with soft silicone. This new soft silicone claw surface has accumulated more than 14,000 grasp attempts on land (71.4%) and underwater (28.6%). The experiments first selected the optimal design through land experiments. Subsequently, the underwater capability was further tested. It was verified that the bionic finger surface design could increase the grasping success rate by up to 18.2%. In summary, a certain number of studies have been performed on the wrist joint, fingers, and finger surface type of claw. Different types of wrists, fingers, and contact surfaces will produce different claw forms and application effects.

**FIGURE 5:**
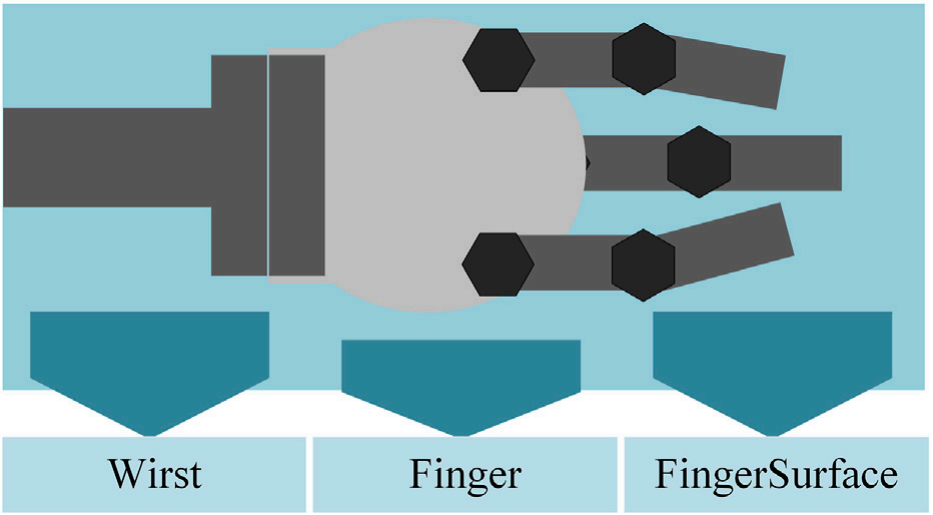
Basic parts of the gripper.

In this chapter, some of the following shaped claws are taken as an example, classified by wrist joints, fingers, and contact surfaces to discuss their respective application fields and the testing environment, as shown in Figure 5.

### 3.2 Cage claws

For example, in the aspect of cage claws, Capalbo Enrico et al. (2022) used rigid claw wrist and cage-shape fluid, flexible fingers to squeeze the internal pad to form some envelope surfaces that fit the grasping object in order to achieve the fine holding function of parallel claw and envelope function of cage claw. In addition, its inner liner (the finger surface) uses an antagonistic hydraulic cylinder to match the volume loss of the liner, and this mechanism has undergone a certain amount of underwater testing in the laboratory.

**FIGURE 6:**
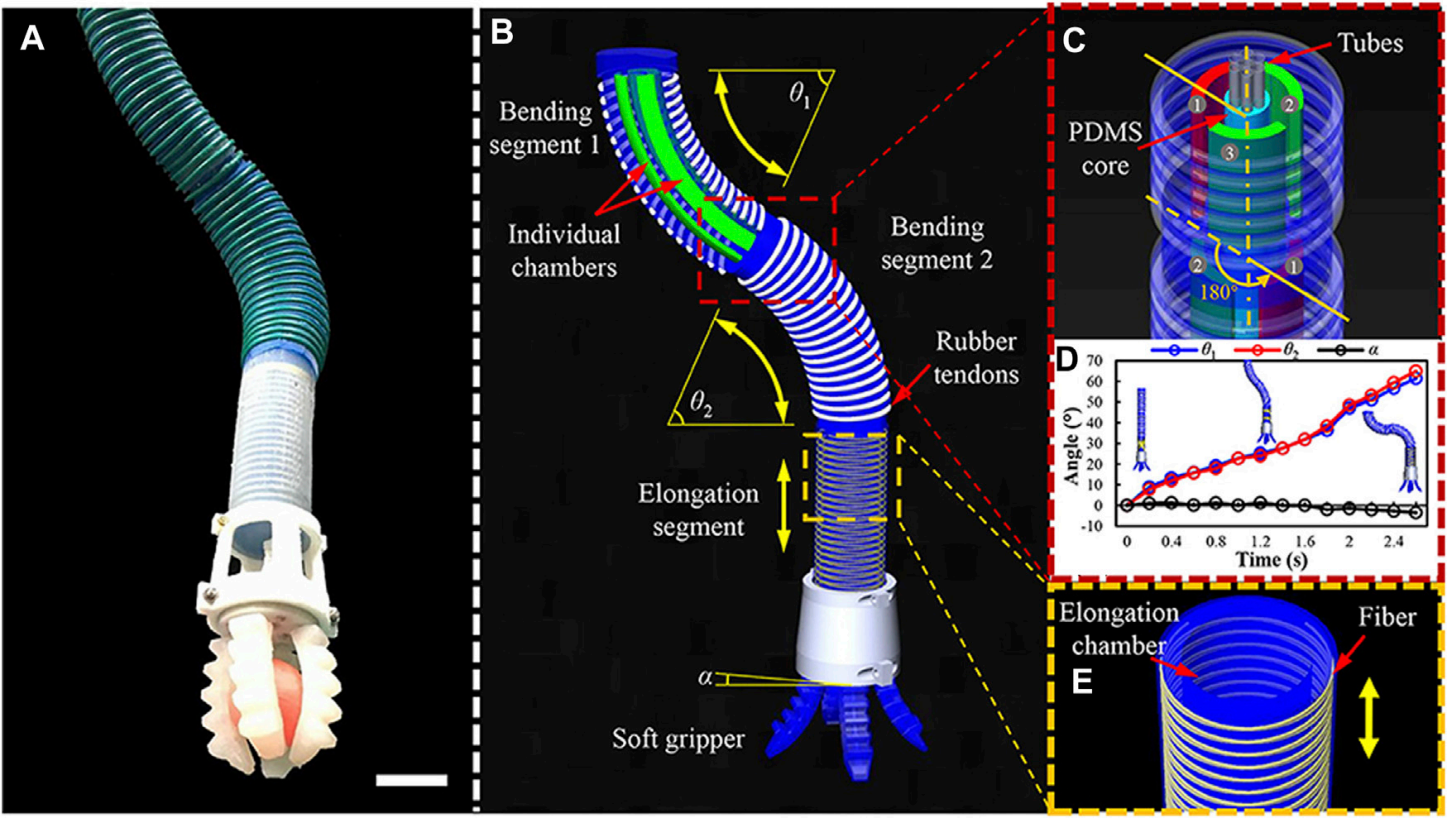
Design and principle mechanics of the underwater soft manipulator. **(A)** An overall side image of the underwater soft manipulator (scale bar 50 mm). **(B)** The underwater soft manipulator is an applied, modularized design consisting of two bending segments: an elongating segment and a soft gripper. θ1 and θ2 represent the bending angles of the two bending segments, and α represents the horizontal angle of the manipulator tip. The manipulator is actuated with an opposing curvature, where θ1 = θ2 and α = 0. **(C)** The two bending segments had a joining angle of 180°. **(D)** θ1, θ2, and α are verified in one actuation with opposing curvature. The two bending angles (θ1 and θ2) are almost equal, and the horizontal angle (α) is zero at each moment. **(E)** Fiber-reinforced elongating segment. The yellow arrow indicates the direction of elongation [reprinted with permission from Gong et al. (2019)].

Earlier studies (Gong et al., 2019; Gong et al., 2018b; Gong et al., 2021) have investigated a 3-DoF soft mechanical arm for fragile grasping in shallow water space using a flexible pneumatic wrist with a reverse bending structure, a cage-type flexible pneumatic gripper, and a silicone gripper surface, as shown in Figure 6. The team first studied the trajectory and working space of the gripper and then conducted real-time closed-loop pickup and placement experiments of the manipulator using a binocular and hand-held camera. The experimental results showed that the claw could successfully collect eight sea thorns and one sea cucumber within 20 min.

**FIGURE 7:**
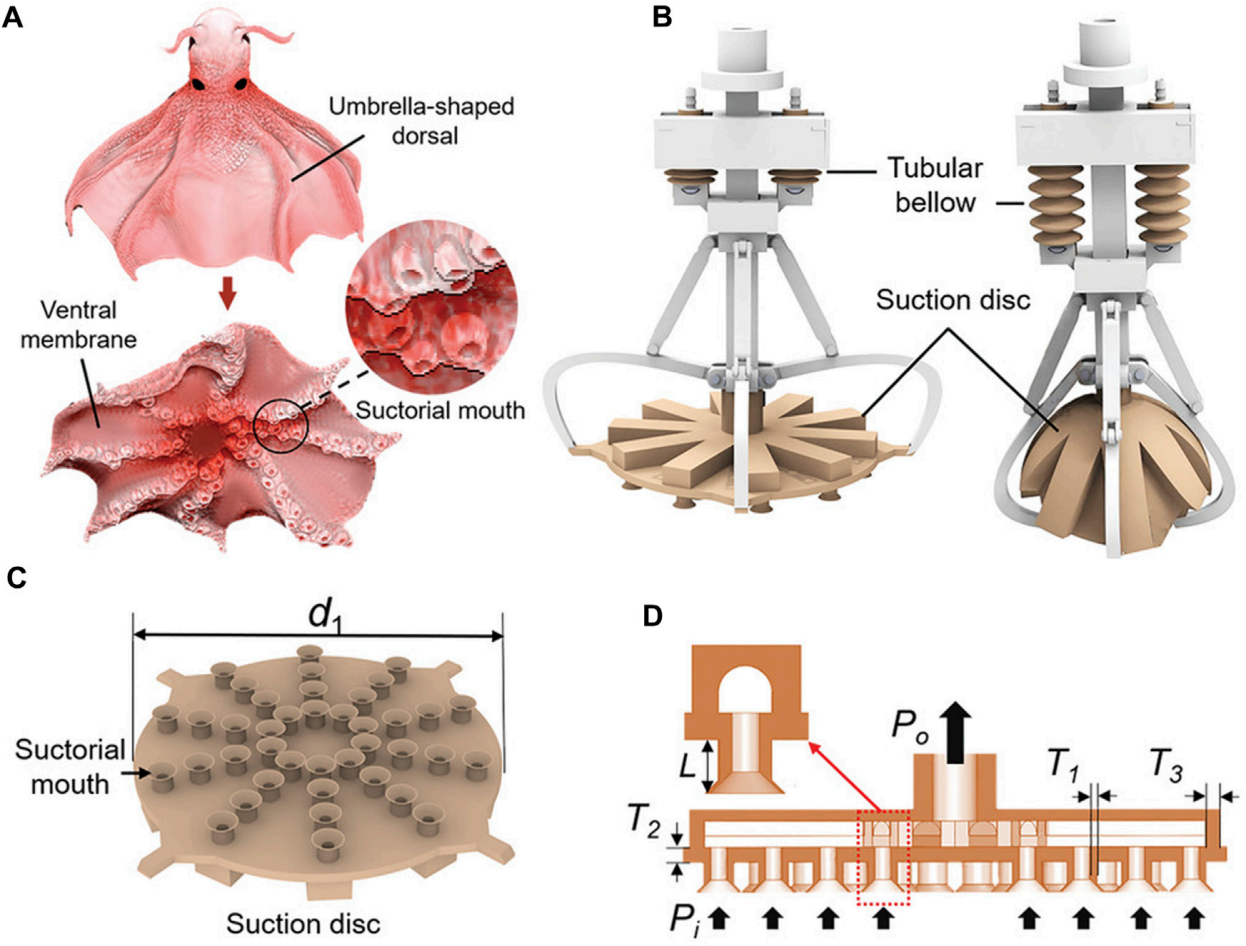
Glowing sucker octopus (*Stauroteuthis syrtensis*)-inspired suction disc. **(A)** Morphology structure of *Stauroteuthis syrtensis*. Suctorial mouth arrays are distributed on the soft arms, and membranes connect the arms to form a disc. **(B)** CAD model of the biomimetic soft gripper. The suction disc can be opened and closed under the drive of the tubular bellow. **(C)** CAD model of the suction disc. Suctorial mouth arrays with funnel-shaped ends are distributed on the suction disc. **(D)** Schematics of the suction disc [reprinted with permission from Wu et al. (2022)].

Wu et al. (2022) introduced a cage-type gripper that is combined with a flexible hydraulic claw wrist and a flexible hydraulic claw finger. Its touching surface is a silicone sucker with adhesive ability. The design of the claw was inspired by *Stauroteuthis syrtensis*. The feature of the gripper is very distinct. When the gripper contacts the underwater target object, the local suction port on the array sucker is blocked, which changes the flow rate in the array suction port to detect whether an object is grasped, as shown in Figure 7. The gripper is ideal for situations where cameras cannot be used, such as murky underwater environments. In addition, the adhesive and grasping of objects (such as turtles) were tested in the underwater environment of the laboratory.

Licht et al. (2016) and Licht et al. (2017) developed a more abstract cage claw, in which the holding wrist is rigid, but the gripping finger and finger surface are composed of a latex balloon filled with a mixture of fresh water and glass beads (approximately 200 µm in diameter). The claw mainly relies on the deformation of the latex balloon to grip a specific object, which is equivalent to an infinite number of fingers for cage grasping. Experiments conducted at sea prove that the gripper can be used for deep-sea grasping missions of more than 100 atm.

### 3.3 Intermeshed claws

In terms of the intermeshed claw, Galloway et al. (2016) and Vogt et al. (2018) designed a gripper with a rigid claw wrist, a hydraulically flexible intermeshed claw finger, and a sponge claw surface. The fingers were typical corrugated claw fingers and were equipped with two sets of replaceable and disassembled fingers, which were convenient for twisting or intermeshing grasping. The final test was mainly conducted on site sampling of specimens, such as coral.

Sinatra et al. (2019) proposed an ultra-flexible soft robot actuator that mainly consists of a rigid claw wrist, a hydraulically driven ultra-flexible intermeshed claw finger, and a nanomaterial finger surface. The grasping test was conducted in the laboratory and aquarium. The main content of the test was to grasp delicate gelatinous marine biological specimens such as jellyfish under the sea. A series of assessments were performed to test the collection area of the claw and the robustness of the claw against external forces.

### 3.4 Parallel claws

**FIGURE 8:**
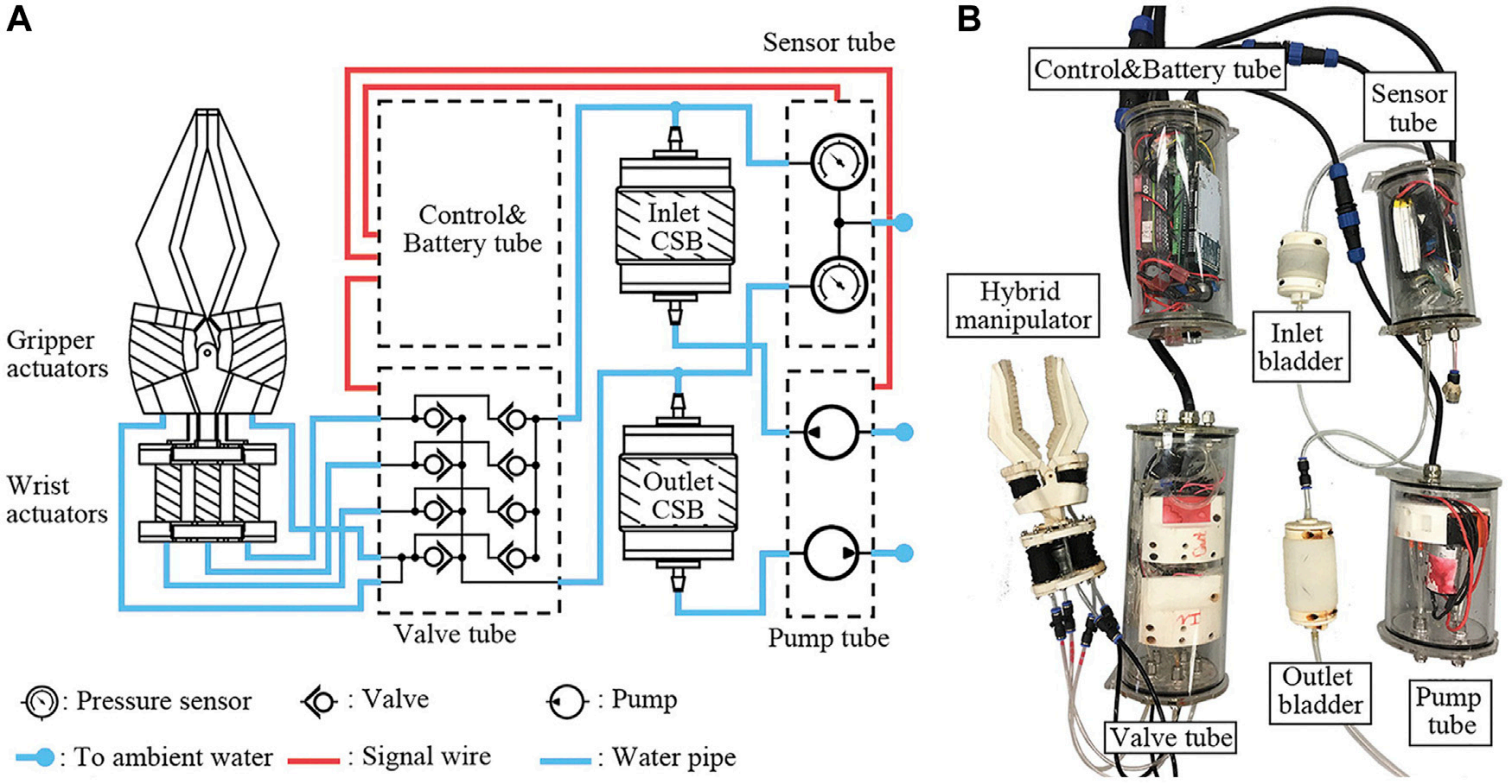
System overview. **(A)** Schematic drawing of the proposed underwater hydraulic system. **(B)** Underwater hydraulic system with the manipulator. Color images are available online [reprinted with permission from Shen et al. (2020)].

In the study of parallel claws, Shen et al. (2020) proposed a design and control method for the underwater robotic arm, whose main structures include a hydraulic wrist, parallel claws combined with rigid and soft materials, and a soft finger face, as shown in Figure 8. The main feature of the manipulator is that it uses a rigid frame and a soft driver as the finger of the claw, which has formatted a soft manipulator prototype with 4-DoF and 15 N load. The gripper conducted an underwater test in the laboratory.

### 3.5 Challenges and limitations

At present, the underwater application of comparison grippers with different structures is mainly for sample collection, including but not limited to the sampling of jellyfish, Echini, and coral samples. For different grippers, the development direction is limited by the application field. As shown in Table 2, the comparison is as follows.

**TABLE 2:** Comparison between different gripper structures.

Structure	Wrist	Finger	Finger surface	Feature	Advantage/limitation	Test environment	Reference
Cage claw	Pincer drive cylinder (rigid link)	Rigid pincer arm	Jamming pad with a fiber-reinforced actuator	Securely grasp objects with a wide range of weights, sizes, and geometries	Good ability to grasp irregular-shaped objects/sacrificed some accuracy of parallel gripper and grasp ability in small objects	Laboratory	Capalbo Enrico et al. (2022)
Cage claw	Rubber tendons	Soft gripper (four fingers)	Rubber surface (shore A hardness of 30)	Could follow complex trajectories, and the tracked points continued to match the programmed path well with an error >13.4 mm	Low-inertia properties of the OBSS soft manipulator/low actuation response speed and grasping efficiency	Underwater (10 m)	Gong et al. (2019); Gong et al. (2018b); Gong et al. (2021)
Cage claw	Tubular bellow	Suction disc	Suctorial mouth	Sensing ability of the suction disc	Can efficiently sense and grasp target objects with various dimensions and shapes/pressure leakage issue	Laboratory	Wu et al. (2022)
Cage claw	Support structures made of light-curing resin	Conveyor belts driven by geared motors	Soft paddles	Rigid and flexible swallowing system	Grasping fault tolerance and structural fault tolerance. The swallowing force is greatly affected by the size of the object	Laboratory	Li et al. (2023)
Cage claw	Silicone personification palm	Bellows-type bending actuators	Filament winding	Bidirectional underwater grasping	The extended workspace provides fault tolerance for grasp. Is challenging to employ the minor open and close motions	Laboratory	Wang et al. (2022b)
Cage claw	Shaft collar	Bearing cylinder	Latex balloon	Better gripper performance can be achieved at higher ambient pressures	With no actuated mechanical components. The strength of the membrane, given the particle composition used	Underwater (1,200 m)	Licht et al. (2016); Licht et al. 2017)
Intermeshed claw	Hydro-Lek wrist clamp	Bellow soft actuator	Memory foam	Replaceable cutting blades and alternative fingers	In suit measurement to deep reef organism	Underwater (100–170 m)	Galloway et al. (2016); Vogt et al. (2018)
Intermeshed claw	PolyJet palm chassis (Rigid Link)	Silicon chamber (Dragon Skin 20)	Nanofiber sheet (Nylon)	Resistant to corrosion in salt water	Low contact pressure (0.0455 kPa)/ larger actuators may require thicker nanofiber sheets	Laboratory	Sinatra et al. (2019)
Parallel claw	Wrist actuators (Dragon Skin 10) and a ball joint	Gripper actuator (Dragon Skin 10)	Soft texture	A consumer-grade ROV could mount the manipulator	Wide grasping range (85 deg.), accurate grasping both onshore and underwater/15 kg, reduce 37.8% speed of the consumer-grade ROV	Laboratory	Shen et al. (2020)

Compared with other claw shapes, the parallel claw is better for the precision grasp of small objects and more suitable for capturing steady or low movement targets. In other words, the moving object is unsuitable for a parallel claw to grasp, for example, catching a moving jellyfish. However, the parallel claw is inevitably an important component for delicate grasping. The further challenge is to improve the ability to grasp and precision in a complex environment.

As a regional capturing gripper, the cage claw is limited by its own envelope structure, and its grasp performance for small objects is not as fine as that of the parallel claw (Herrero-Pérez and Martínez-Barberá, 2022; Songlin et al., 2017). However, its regional capture ability for small marine creatures (e.g., moving fish) has shown unique advantages. The next step of this type of claw is improving the ability of the grasp area and the efficiency of grasping.

Similar to a claw, the intermeshed claw has a certain grasp area and a certain grasp accuracy that has a unique advantage, such as grasping for stick-like objects. Its intermeshed claw fingers make it easier to grasp and hold the grasped object. Furthermore, their fingers are not likely to interfere. Although the intermeshed claw has the advantages of cage and parallel claws, the design of the structure highly depends on the grasping object. When the grasping object is unknown, the ability to grasp declines sharply. The problem has become a hot spot in intermeshed claw research.

## 4 Soft actuator

Among the diversity of marine life, animals walk in different ways. Like most fish in the ocean, they swing by bending their bodies to provide a driving force in the water (e.g., swing locomotion). The octopus and jellyfish are driven by pressure differences (e.g., jet drive). Additionally, starfish and sea spiders like to walk underwater or on the surface. Among them, swing locomotion drives are divided into BCF locomotion (e.g., most fish and water snakes) and MPF locomotion (e.g., manta rays) (Raj and Thakur, 2016).

### 4.1 Locomotion

#### 4.1.1 BCF locomotion

**FIGURE 9:**
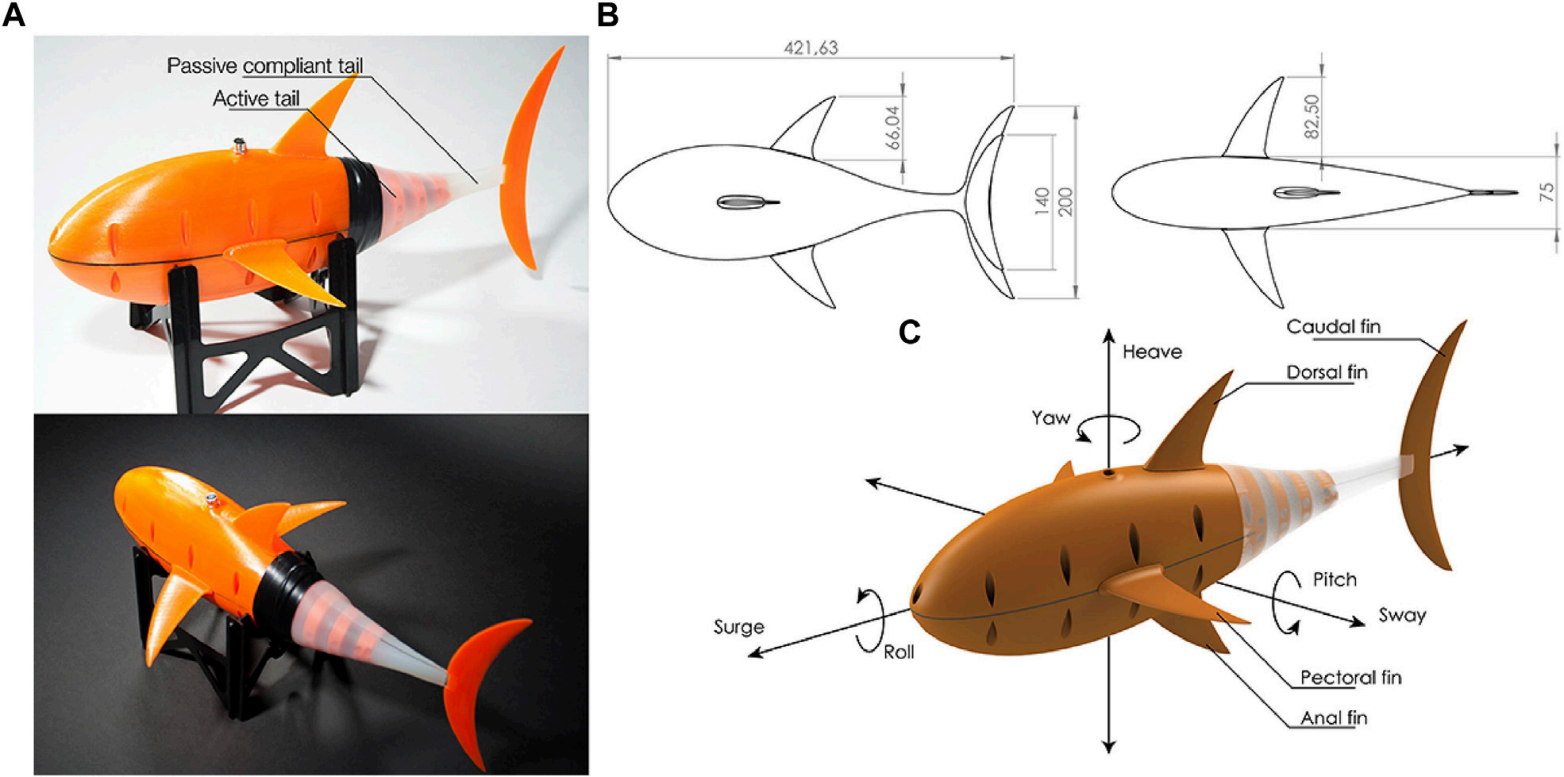
**(A)** Design of the open-source soft robotic fish. **(B)** Side and top views of the soft robotic fish with its main dimensions; **(C)** explanation of the terms used for fish anatomy and fish stability [reprinted with permission from Berg et al. (2022)].

As the fish that people have been in contact with and understood for the longest time, research on swing locomotion has also made great progress (Wolf et al., 2020). In the research direction of BCF locomotion, some scholars (Berg et al., 2022) modeled the thunniform swimming mode and introduced an open-source soft robotic fish that achieves a maximum speed of 0.85 m per second using a propulsion system that can adjust the shape of the sine wave to achieve a higher oscillation frequency, as shown in Figure 9.

Katzschmann et al. (2018) and Katzschmann et al. (2016) used water as the transmission fluid to drive the swing of the soft fish tail through the water circulation in the internal waterway channel. In order to achieve the purpose of swimming, the main energy supply mode was using a gear pump to promote the water pressure. In addition, a camera, an underwater wireless transceiver, and pitching fins are added to the propulsion frame to control the pitching and swimming movements of the whole fish.

Marchese et al. (2014) focused on the fast escape response of soft fish. Under the premise of having all the subsystems of the traditional robot (power, drive, processing, and control), the experiment of simulating the escape of bionic fish was performed. The kinematics and controllability of the robot during the simulated escape response were analyzed and compared with those of the biological fish. In conclusion, the soft robot has an input–output relationship similar to that of the biological fish during the escape response.

**FIGURE 10:**
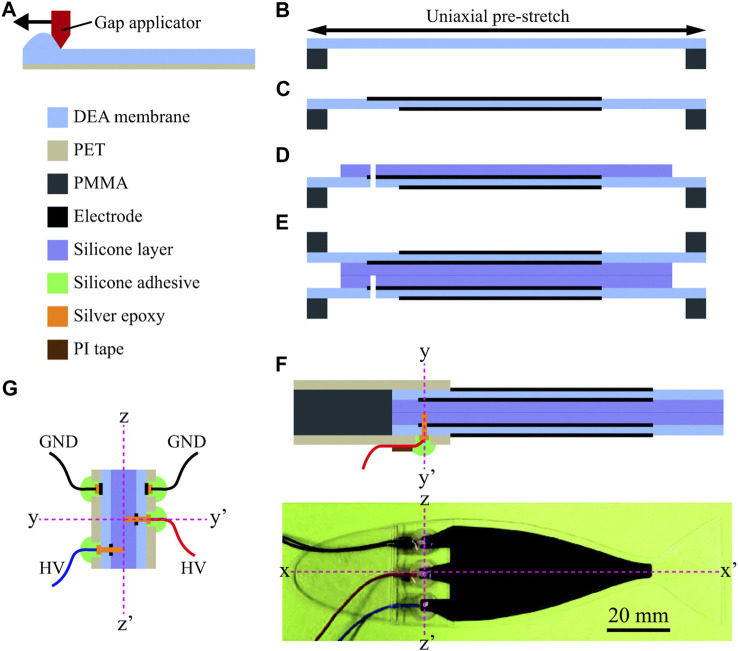
Fabrication process of the robot. **(A)** DEA elastomer is blade casted on a PET film and **(B)** stretched uniaxially. **(C)** Electrodes are patterned on the stretched membrane. **(D)** Body layer made of silicone elastomer is bonded, and a punched hole is made. **(E)** Bonding two-half samples. **(F)** Attaching the head part and wiring. **(G)** Alignment of the electrode connections. PET, polyethylene terephthalate [reprinted with permission from Shintake et al. (2018)].

Shintake et al. (2018) developed a fish robot with a length of 150 mm, a thickness of 0.75 mm, and a weight of 4.4 g using dielectric elastomer actuators (DEAs) to mimic the swing of fish swimming. The robotic fish swims by body, BCF locomotion, or both and is made using laminated silicone layers and two counter-configured DEAs. Only two DEAs should be driven to produce waves, thus achieving a fish-like motion, as shown in Figure 10. The design was guided by a mathematical model based on Euler–Bernoulli beam theory and considered the heterogeneous geometry of the robot and the hydrodynamic effect of water. By comparing the modeling results of the robot fish with the experimental results, it is concluded that the measured peak frequency of the thrust generated by the robot is similar to the natural frequency calculated by the model. At 0.75 Hz, the peak swimming speed of the robot is 37.2 mm/s (0.25 body length/s), which swims much like a real fish.

Zhang et al. (2018) used an electromagnetic drive to build ostraciiform robots. A two-segment caudal fin design was used, which is a substantial improvement over the existing single-segment caudal fin. The proposed two-segment fins help reduce drag force, improve resonant frequency, and achieve a wide range of controllable thrust. Six types of caudal fins were compared in the overall experiment, more than 1,200 tests were designed, the resonance frequency was nearly three times higher than that of the single-segment homogeneous caudal fin, and the thrust was 12% higher. It is also proved that the peak frequency can be selected by customizing the tail fin according to the application.

Zhu et al. (2019) introduced a platform (Tunabot) by mimicking *Thunnus albacares* and *Scomber scombrus* to study the high-frequency swimming performance of fish. The kinematics, speed, and force of the body were measured to quantify swimming performance and study the flow field generated by the tail while increasing the tail beating frequency. The Tunabot (255 mm long) can achieve a maximum tail-clap frequency of 15 Hz. The swimming speed is equivalent to four body lengths per second and has a range of 9.1 km if it swims at a speed of 0.4 m/s. Although the swimming speed is 1.0 m/s, the range is 4.2 km (assuming a 10 Wh battery).

#### 4.1.2 MPF locomotion

The aforementioned study describes how swaying from BCF locomotion can provide a certain amount of power to swim, and this is the swaying structure adopted by most fish. However, in addition to BCF locomotion, MPF locomotion can drive organisms to swim. For example, Li et al. (2021) developed a soft animatronic fish with a fully integrated onboard power supply and remote control system. Driven entirely by a soft electroactive structure composed of dielectric elastomers and ionic conducting hydrogels, the fish swam at a speed of 6.4 cm/s (0.69 body length per second) and behaved stably over a wide range of temperatures. Because of its transparency, it can be used for stealth navigation.

Similarly, Li et al. (2017), inspired by the structure of deep-sea snails and fish, developed a tethered soft robot for deep-sea exploration. Its onboard power, control, and driving electronics are all integrated into the silicone matrix to avoid the influence of pressure. The dielectric elastomer material used in the robot’s flapping wings was carefully designed to enable the robot to launch successfully during field tests at 10,900 m deep in the Mariana Trench and to swim freely at 3,224 m deep in the South China Sea. The research work highlights the potential to design soft and lightweight equipment for extreme conditions.

### 4.2 JetDrive

Cuttlefish and jellyfish use jets to exert a reaction force on themselves to swim through the water, which means that in addition to some creatures that swim through water using oscillations, some creatures swim through water using the jet drive. At the same time, this inflow mode has inspired many scholars to study jet flow, such as Bi and Zhu (2021), who studied the influence of nozzle geometry on system performance during a single discharge at a low Reynolds number. The force decomposition algorithm was used to decouple thrust into three parts: normal stress at the exit, jet flux, and time derivative of internal fluid momentum. The paper concluded that the stroke ratio and deformation time history were fixed. In this case, the total thrust generated by the system was mainly determined by the nozzle outlet size.

Based on the open-source platform (OpenFOAM), Zhang et al. (2020) adopted the CFD method to solve the efflux fluid and conducted theoretical research, mainly studying vortex ring evolution and propulsion efficiency characteristics. In the first part, the pulse process of different strokes was simulated and compared with the standard experiment. In the second part, a reasonable open-water property calculation method was proposed to study the vortex ring thruster (VRT). The thrust coefficient and propulsive efficiency were obtained. The results showed that the high efficiency of VRT was close to 1 at a higher propulsion rate. In addition, the efficiency of four asymmetric velocity schemes and three symmetrical velocity schemes was compared. The final results showed that if the pulsation effect was prominent, a smaller stroke could achieve greater efficiency.

Cheng et al. (2019) described a tethered soft robot jellyfish with high maneuverability, which could imitate the performance of natural jellyfish. The prototype electrode is made of carbon grease sandwiched between two thin dielectric elastomer films. The frame of the material is made of silicone elastomer with six plastic paddles attached to it. The robot jellyfish recorded a maximum swimming speed of 1 cm/s and a peak thrust of 0.00012 N. A finite element simulation was developed to theoretically study the performance of the robot jellyfish. By embedding a compact remote-controlled power source, the robotic jellyfish is autonomous. In this case, the maximum peak velocity is approximately 0.5 cm/s. The working principle of the bionic robot jellyfish can be used for the field of research and guide the design of soft robots and flexible devices.

Inspired by jellyfish creatures, Christianson et al. (2019) connect a ring of rimless DEAs to the non-expandable layer to generate a single structure curved toward the passive side to generate power stroke and effectively restore the original structure when the robot glides. The flexible rimless DEAs can use fluid electrodes to apply a voltage to the film and can achieve efficient movement of the eel robot without the need for a rigid frame. The average speed of the soft robot swimming is 3.2 mm/s. This work demonstrated the feasibility of using DEAs with fluid electrodes for low-power, silent operation in underwater environments.

The jellyfish machines of Frame et al. (2018) are pneumatically driven, with eight pneumatic network tentacle actuators extending outward from their centers. These jellyfish robots can swim and turn in the ocean. Moreover, jellyfish can pass through pores narrower than the nominal diameter of jellyfish. Each of the five jellyfish robots has a different body composition, tentacle actuator, and shore hardness to facilitate the study of these three factors and determine the influence of the tentacle stroke on the measured thrust. All three factors significantly affect average thrust generation, and the half-stroke drive amplitude peaks at a frequency of 0.8 Hz.

Godaba et al. (2016) mainly studied a jellyfish robot with DEA, which has muscle-like characteristics, such as large deformation and high energy density. The deformation and force of the actuator were experimentally tested. The performance of the actuator was analyzed by theoretical simulation, and the results were qualitatively consistent with the experimental results. Preliminary studies have shown that the jellyfish robot, based on dielectric elastomer technology, can move effectively in water. The robot also exhibits fast response and high load capacity (relative to its self-weight)

Wang Tao et al. (2019) studied the ability of pulse-jet underwater soft robots to turn using a steerable nozzle through experiments. The drive of the robot is based on the periodic conversion of slow-charged elastic potential energy into fluid kinetic energy to perform a cyclic pulsed jet. A steerable nozzle is added at the end of the jet pipe of the robot to deflect the jet in order to conduct thrust vector control. The results show that the drive scheme has a higher control ability from static to starting. The turning radius is approximately half the bionic body length, and the optimal nozzle deflection angle is 35°. The most important factor affecting the turning efficiency is the fluid momentum loss caused by nozzle deflection.

Wang Yuzhe et al. (2022) developed a completely transparent soft-bodied jellyfish robot, which is transparent and can move in all directions. The robot is driven by transparent dielectric elastomer drivers (DEAs), a hybrid silver nanowire network, and conductive polymer poly(3,4-ethylenedioxythiophene):poly(styrenesulfonate)/waterborne polyurethane as a compatible electrode. The electrode exhibits a great tensile property, low stiffness, high transmittance, and excellent electrical conductivity under large strain. Therefore, with the very high-bond film as the medium layer and polydimethylsiloxane as the surface coating, the maximum area strain of the highly transparent DEAs based on the hybrid electrode can reach 146%, and the hysteretic loss is only 3%. Driven by these transparent DEAs, the soft jellyfish robot can achieve vertical and horizontal movements in water by imitating the actual pulsating rhythm of *Aurelia aurita*.

Zhang et al. (2021) used a biomimetic soft-robotic siphon (BSRS) as the propulsion unit, which was composed of a new central flow regulative duct (CFRD) surrounded by three siphon actuation muscles (SAMs). Hydraulic pressurization of SAMs enables thrust vectoring by deflecting the BSRS and flow regulation by proportionally alternating CFRD ports. Flexural deformation and flow regulation experiments were performed using the BSRS prototype. The results show that the bending range of the BSRS is more than 180°, and the current limiting capacity is up to 100%. The robot achieves a burst effect by exceeding a constant flow rate of up to 50%, achieving a huge thrust increase in a very short time. This work demonstrates the feasibility of combining omnidirectional deflection with flow regulation in a soft robot mechanism, paving the way for compact water jet propulsion of underwater vehicles.

### 4.3 Crawling/floating

In terms of unptderwater crawling, Ishida et al. (2019) developed an underwater crawling robot whose top and legs could be inflated and deflated. The change in body shape affects its hydrodynamic properties. When the robot (2.87 N underwater weight) needs to stay still in the flow, an asymmetrical body is more resistant to body sliding. Body shape had a significant effect on the robot’s ability to walk upstream, with uninflated bodies able to walk in a flow of 0.09 m/s, but larger inflated bodies were pushed backward. The paper demonstrated that such robots can detect changes in velocity using commercial flow sensors and respond by deforming into a hydrodynamically preferable shape.

Inspired by starfish, Patterson et al. (2020) proposed a mobile cordless underwater crawling soft robot (PATRICK). PATRICK consists of five flexible legs driven by 20 SMA wires, providing a wide variety of motion possibilities through its large input space. The experiment shows that the robot can be ordered to move to the target state using the planning mode. These experiments provide examples of closed-loop state space target searching of tethered underwater flexible crawling robots, and some progress has been made in fully autonomous soft mobile robot systems.

**FIGURE 11:**
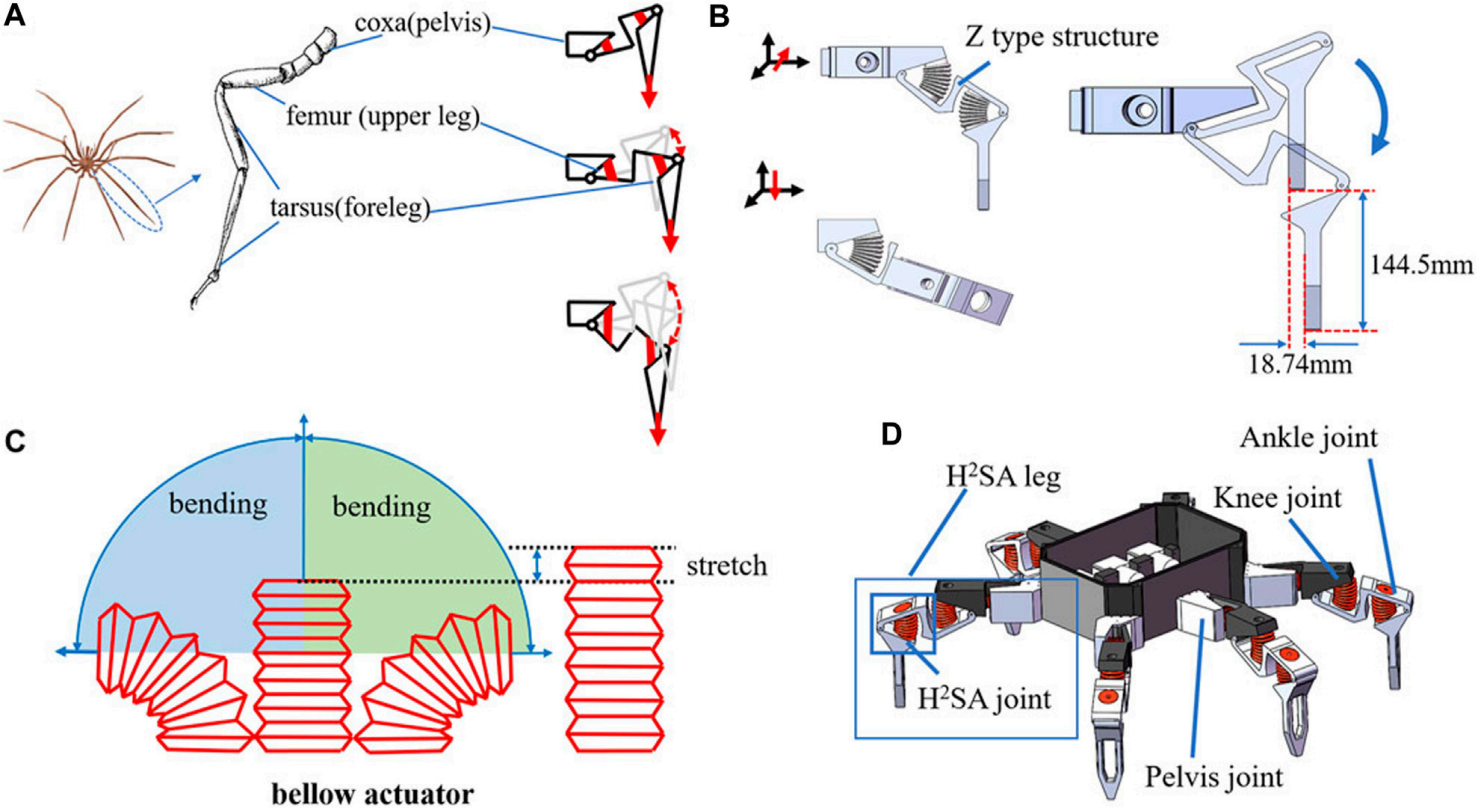
Concept of soft underwater crawling robot: **(A)** illustration of one-leg structure and gait; **(B)** single-leg 3D model; **(C)** movement characteristics of bellows actuator; **(D)** conceptual design of a proposed soft underwater crawling robot [reprinted with permission from Tan et al. (2021)].

Inspired by sea spiders, Tan et al. (2021) proposed a soft robot design method for underwater crawling robots, as well as a rigid-flexible hybrid multi-joint leg design. The joint has a quasi-linear range of motion, and at the same time, the inherent flexibility of the soft actuator is utilized to maintain good passive impact compliance. Figure 11 shows the overall and joint structures of the flexible robot.

Huang et al. (2021) introduced a model based on discrete differential geometry to solve the nonlinear deformation problem when the soft material structure interacts with the liquid environment in terms of water walking. An untethered omnidirectional star swimming soft robot is designed by imitating the structure of a starfish. The robot has been tested to be able to move with multiple swimming gaits. The quantitative agreement between experiment and simulation demonstrates the potential application of this numerical model in robot design and model-based control schemes.

**FIGURE 12:**
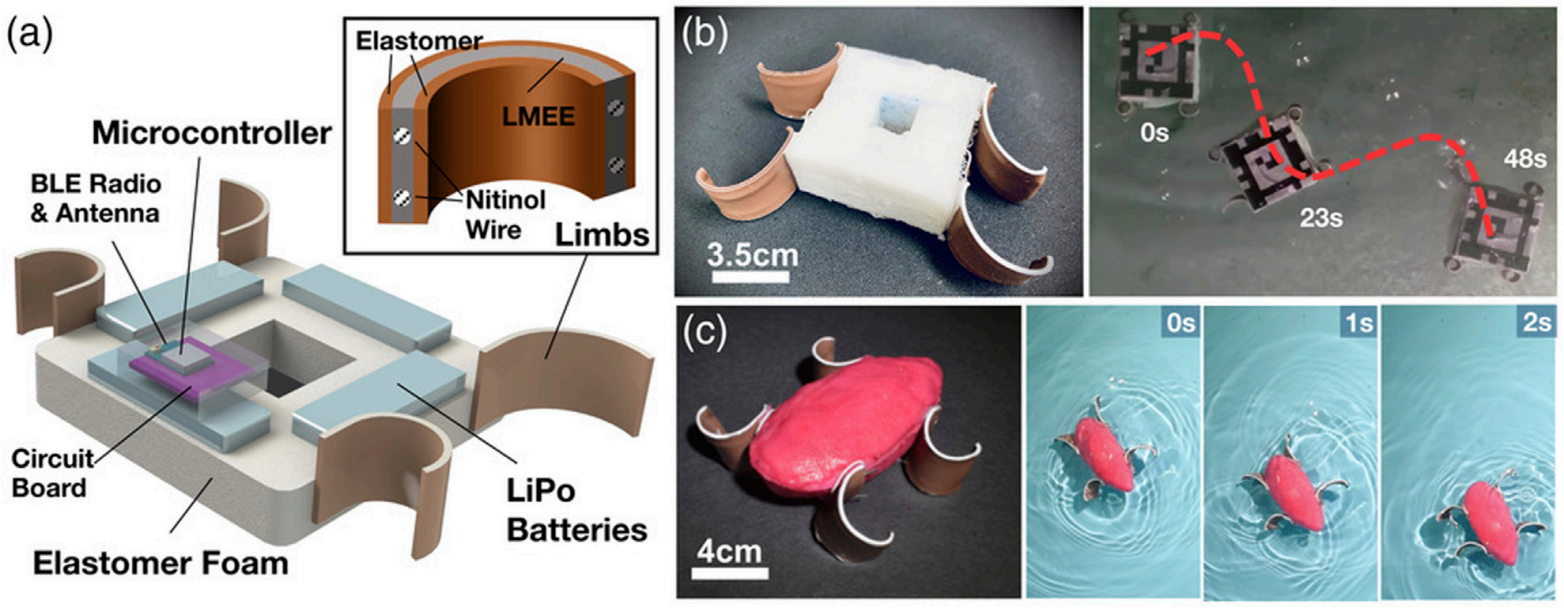
Design and geometry of the untethered frog-inspired soft robot. **(A)** Components of the robotic system. **(B)** Square-shaped robot for examining motion along a curvilinear path. **(C)** Streamlined robot for >1 blps swimming along a straight line. The square-shaped robot is used in the simulation pipeline so that 2D simulations can be performed. The streamlined robot is intended to show the potential performance of such swimming robots with a design that minimizes drag [reprinted with permission from Huang et al. (2022)].

Moreover, on this basis, Huang et al. (2022) studied a completely cordless soft frog swimming robot framework based on physical modeling, motion planning, and control. They used the discrete elastic rods (DERs) physics engine to disperse the soft robot into many stretchable and flexible rods. In terms of hardware, an untethered water soft robot is designed to perform a frog-like rowing behavior, as shown in Figure 12.

### 4.4 Challenges and limitations

Compared with the existing turbine drive, the low swing frequency of the flexible drive is very suitable for the observation of marine biological population activities, and the high energy conversion efficiency (Gupta et al., 2019) is very suitable for underwater endurance operations. Although the benefits are enormous, part of the robot is made of flexible materials with a complex underwater environment, which has raised concerns about its stability and durability. These problems also need certain research and development to handle. The related progress is shown in Table 3.

**TABLE 3:** Comparison between different actuator structures.

Structure	Speed	Driven period	Feature	Advantage/limitation	Driving force	Test environment	Reference
BCF locomotion	850 mm/s (2.02 body length/s) (max)	5.46 Hz	Using the continuous rotation of a DC motor to pull the cables connected to both sides of the active tail segment	Using active tail segment and passive tail segment/exceed the predictions from a tailbeat frequency of approximately 2.5 Hz	Pulling cables	Laboratory	Berg et al. (2022)
BCF locomotion	217 mm/s (0.5 body length/s at depths of 0–18 m) (avg.)	0.9–1.4 Hz	The on-board capabilities of an untethered mobile underwater observatory	Tested at coral reefs 18 m depths/low swimming efficiency, limited depth range	Hydraulic actuation	Underwater (18 m)	Katzschmann et al. (2018); Katzschmann et al. (2016)
BCF locomotion	150 mm/s (0.44 body length/s) (avg.)	1.67 Hz	Using gas pump to reserve energy and having escape ability	Having the escape response/the gas delivery system is responsible for considerable resistive energy losses	Pneumatic actuator	Laboratory	Marchese et al. (2014)
BCF locomotion	37.2 mm/s (0.25 body length/s) (max)	0.75 Hz/5 kV	Only 4.4 g of weight and using DEAs as a main driver	The robot resembles real fish and displays a Strouhal number very close to that of living fish/the feature of the structure may change by the oxidation	DE actuator	Laboratory	Shintake et al. (2018)
BCF locomotion	300 mm/s (2 body length/s) (max)	14 Hz	Segmented caudal fin, achieved 70 Hz operating frequency	Peak frequency could be selected using fin design/external power supply and control electronics via tether	Solenoid actuator	Laboratory	Zhang et al. (2018)
BCF locomotion	1,020 mm/s (4 body length/s) (max)	15 Hz	Swims at 0.4 m/s and has a range of 9.1 km, swims at 1.0 m/s and has a range of 4.2 km (assuming a 10 Wh battery pack)	Low cost of transport/external control signal via tether and lacks control surfaces to adjust turns in yaw or changes in pitch	DC motor trans to lateral linear movement	Laboratory	Zhu et al. (2019)
MPF locomotion	51.9 mm/s (0.45 body length/s)	1 Hz/8 kV	Lightweight DE actuators, snailfish-like	Decentralized electronics and DE-driven flapping fins/needs high-voltage amplifier	DE actuator	Underwater (10,900 m)	Li et al. (2021)
MPF locomotion	64 mm/s (0.69 body length/s)	5 Hz/8 kV	Self-powered soft robots with high mobility, environmental tolerance, and long endurance	Untethered electronic fish, excellent environmental adaptability and disguising performance/need high-voltage amplifier	DE actuator	Laboratory	Li et al. (2017)
JetDrive	10 mm/s (max)	1.6 Hz/9 kV	Jellyfish-like	Low cost and easy assemble/low anti-interference performance	DE actuator	Laboratory	Cheng et al. (2019)
JetDrive	Untethered: 3.2 mm/s (avg.),7.1 mm/s (max)	0.2 Hz/7 kV	Jellyfish-like, low cost of transport (30), 2.7 h actuation (180 mAh)	Swim bladder equipped to provide buoyancy control/incapable of providing large forces	DE actuator	Laboratory	Christianson et al. (2019)
JetDrive	/	0.8 Hz (half-stroke)	Best performance, tentacle actuator-flap material Shore hardness composition of 30–30	Squeezes through narrow conduits, swims directionally by temporally offsetting tentacle actuation strokes on opposing sides of the robot/the horizontal motion of the jellyfish was not directly controlled	Hydraulic/pneumatic actuators	Underwater (5 m)	Frame et al. (2018)
JetDrive	Rigid, tethered	Rigid, tethered	Rigid bell with the dielectric elastomer actuator	Fast response and high capacity of payload external power supply and control electronics via tether/was not directly controlled	DE actuator	Laboratory	Godaba et al. (2016)
JetDrive	295 mm per release	15 s (inflate), 0.5 s (eject period)	Change the periodic conversion of slowly charged elastic potential energy into fluid kinetic energy, cephalopods’ propulsion	Short range, highly maneuverable/ significant viscous losses due to the sharp corners within the nozzle conduit	Hydraulic actuation	Laboratory	Wang et al. (2019c)
JetDrive	5.4 mm/s (avg.)	2 Hz/5 kV	Using hybrid silver nanowire networks, has large stretchability, low stiffness, high transmittance, and excellent conductivity	Transparent DEA and can achieve maximum area strain of 146% with only 3% hysteresis loss/jamming by the dragging force, tethered	DE actuator	Laboratory	Wang et al. (2022c)
JetDrive	Rigid, tethered	Rigid, tethered	A novel central flow-regulative duct encircled by three circumferential siphon actuation muscles	Bending range of over 180° and flow-restricting capability of up to 100%/tether was not directly controlled	Hydraulic actuation	Laboratory	Zhang et al. (2021)
Crawling	15 mm/s (still water)	/	An underwater legged robot with soft legs and a soft inflatable morphing body	Able to change body shape against hydraulic flow/lack resistance of the drag on the hydraulic tether	Hydraulic actuation	Laboratory	Ishida et al. (2019)
Crawling	10 mm/s (0.04 bldy length/s) (avg.)	2.52 s (avg.)	Five flexible legs actuated by 20 shape memory alloy (SMA) wires	A wide variety of possible motions/every actuator is a nonlinear dynamical system with a large amount of hysteresis	SMA actuation	Laboratory	Patterson et al. (2020)
Crawling	/	/	Rigid–soft hybrid multi-joint leg with quasi-linear motion range and force exertion	Using rigid structural components to reinforce the flexible soft actuators/complicated production	Hydraulic actuation	Laboratory	Tan et al. (2021)
Floating	2.83 mm/s	/	Untethered omnidirectional star-shaped swimming soft robot	Capable of moving with a variety of swimming gaits/disturb by flow	SMA actuation	Laboratory	Huang et al. (2021)
Floating	48 mm/s (avg.)	1.4 Hz	An untethered aquatic soft robot that performs frog-like rowing behaviors	Limbs can be replaced within seconds/lack of data on manufacturing accuracy	SMA actuation	Laboratory	Huang et al. (2022)
Crawling	5 cm/s (max)	1.4 s (avg.)	The complete absence of rigid parts makes it possible to replicate the high compliance and flexibility of the octopus arm	Pass through confined and unstructured spaces/the control of soft robots through distributed sensors is difficult	SMA springs or cables	Laboratory	Krieg et al. (2015)

In addition, locomotion showed a strong energy conversion advantage and a high movement speed (within a certain swing frequency). However, there was a certain gap compared to the current research on the movement speed, and the endurance and stability should be discussed in a certain number of experiments.

As a swimming mode with intermittent output, Jet Drive can well imitate the swimming of creatures such as octopuses (Krieg et al., 2015). However, its complex turning structure and energy dissipation are unavoidable. At present, the research is mainly focused on the optimization of the overall mechanism and underwater test. Further work could be the movement affected by the external environment, especially under high pressure. It still needs much time for testing in the deep salty water area.

The crawling and floating movements are easier to realize compared to other neutral buoyancy movements. However, this type of movement sacrifices most of the area underwater so that it can only move in a certain place. Despite this limitation, the robot’s carrying capacity and structure stability have made a space in actuation (Ren and Yu, 2021). Further progress could be achieved through structure optimization and control strategy evaluation (Cianchetti et al., 2015).

## 5 Soft sensor

As fish use the lateral lines of their bodies to sense water velocity and vibrations, octopuses use suckers and tentacles to sense the presence of objects. Perception, as an important source of information, prompts people to further develop flexible underwater sensors, such as velocity sensors inspired by the lateral line of fish (Jiang et al., 2016; Liu et al., 2016) and skin sensors inspired by animal skin. Sensors combined with the characteristics of the underwater environment have achieved certain development in recent years (Bora et al., 2018; Han et al., 2018).

### 5.1 Velocity sensors

In the field of hair sensing, Abels et al. (2016) constructed a microelectromechanical system (MEMS) structure to simulate the biological superficial neuromasts found in the lateral organs of fish. Based on the piezoresistive strain principle of the cantilever beam driven along the stress, the local flow velocity was measured to understand the sensitivity to temperature changes and response to changes in the relative flow direction. Furthermore, two cantilever flow sensors with opposite and parallel bending directions (Abels et al., 2019) were designed and compared with a single cantilever hair sensor.

Furthermore, Asadnia et al. (2015) developed an artificial hair cell flow sensor array. The flow sensor adopted polymer hair cells manufactured by stereolithography and mounted on a microdiaphragm with a floating bottom electrode. By installing a polymer tube with pores, external fluid can be directed toward hair cells embedded in the tube. The experimental results show that the vibration of 35 Hz oscillating dipole stimulation has high sensitivity and low detection limits. The flexible array of such sensors is capable of locating using underwater dual stimuli.

**FIGURE 13:**
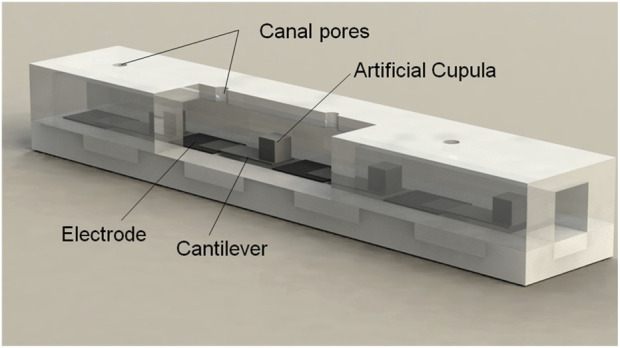
Schematic structure of the proposed ALL canal system [reprinted with permission Jiang et al. (2017)].

Inspired by the artificial lateral system with different conduction mechanisms (Jiang et al., 2019a) in the field of artificial flow sensors, Jiang et al. (2017) developed a cantilever flow sensing element based on polymer materials, formed by lamination of polypropylene and polyvinylidene fluoride (PVDF). The cantilever flow sensing element was integrated into the polydimethylsiloxane (PDMS) pipe, as shown in Figure 13. The sensor has high-pass filtering capability to attenuate low-frequency stimuli, and the pressure gradient detection limit is approximately 11 Pa/m at a frequency of 115 ± 1 Hz. Its structure is flexible and can resist certain interference.

### 5.2 Skin sensors

In terms of underwater flexible contact sensors, He et al. (2022) proposed an adaptive soft sensor whose manufacturing method can be directly used in 3D printing. The whole component is controlled by pneumatic pressure, and the stiffness can be changed within a certain range. The influence of stiffness adjustment on sensing characteristics is studied. The results show that this class of adaptive sensors exhibits good sensitivity, high sensor repeatability, low latency, and good manufacturing repeatability.

Furthermore, Ibarra et al. (2022) used 3D printing technology to print out sensitive, thin, and conductive channels in the soft matrix to construct strain sensors. The effects would also change according to different wiring modes of conduction channels.

Ren et al. (2022) introduced an anti-expansion hydrogel. This hydrogel has high toughness, compressive modulus, ionic conductivity, and, most importantly, anti-expansion properties, with only 9% equilibrium expansion when in water for 30 days. In addition, this research demonstrates some basic application scenarios of this hydrogel as a sensor to better shorten the development cycle of the sensor based on the hydrogel.

### 5.3 Challenges and limitations

As a very specific structure in soft robots (Ma et al., 2019), sensors can perform much work in the underwater environment, which significantly differs on land (Ma et al., 2020).

Due to the differences in sensing media, compared with sensors in dry air, the development of underwater sensors is more inclined to detect low-frequency signals (Zhai et al., 2021), such as the detection of dipoles and the flow rate of the fluid. In addition, sensors on underwater contact forces and other aspects have been further developed.

Compared with the normal sensor, the underwater sensor is characterized by the differences under different working environments, as shown in Table 4. Therefore, certain requirements have been commanded for waterproof performance, detection frequency, and anti-interference ability (Jiang et al., 2019b; Jiang et al., 2020; Jiang et al., 2019a). At present, underwater sensors are mainly based on vibration sensing mimicked by the lateral line structure of fish and pressure sensing modeled on human skin. At this stage, the development has achieved good results, but further experiments are needed to prove its stability, sustainability, and other performance.

**TABLE 4:** Comparison between different types of sensors.

Structure	Test speed	Sensitivity	Feature	Advantage/limitation	Geometry	Reference
Velocity sensors	10∼32 m/s (in air)	40 μV/(m/s) between ±10–20 m/s (in air)	Antiparallel cantilever pairs exhibit an axially symmetrical sensitivity, provides a sinusoidal response	Better compensates for temperature changes. Main limiting factor is the signal-to-noise ratio (SNR)	Bent cantilever (1,500 × 100 × 4 μm)	Abels et al. (2016); Abels et al. (2019)
	Equal to 0.7∼2 m/s (in water)	80 μV/(m/s) between ±20–32 m/s (in air)				
Velocity sensors	0–75 mm/s (in water)	0.8 mV/(mm/s) (in air at 35 Hz)	Cut-off frequency is 10 Hz and has a flat frequency response	Low threshold detection limit (8.2 μm/s in 35 Hz). Fabrication complexities could reduce the surface flatness	Bent cantilever (1,400 × 1,400 × 2,700 μm)	Asadnia et al. (2015)
		22 mV/(mm/s) (in water at 35 Hz)				
Velocity sensors	0–15 mm/s (in water)	1e-6 s (in water at 1 Hz)	The sensing fusion of SNs and CNs	Ultrasensitive and highly accurate flow-sensing abilities. Sensitivity, noise, durability, stability, and sensor fusion methodology face significant challenges	Bent cantilever (5,000 × 2,000 × 86 μm)	Jiang et al. (2019a); Jiang et al. (2017)
Structure	Test force	Sensitivity	Feature	Advantages and disadvantages	Geometry	Reference
Skin sensor	0–50 N	2.6 kPa/N	Pneumatic drive, adjustable stiffness	Low cost, compact size, and ease of integration in soft robotic systems. Height simulation average error	Total structure (10 mm semisphere)	He et al. (2022)
Skin sensor	0∼1 N	9.56 kOhm/mm (0.5 mm diameter)	Mechanical diodes	Sensitive, thin, and conductive channels. The gel cannot be subjected to significant shear stress at the channel’s boundaries	Ink line (13.5 mm sphere surface)	Ibarra et al. (2022)
Skin sensor	/	/	Movements of different parts can be easily identified	High toughness, compressive modulus, ionic conductivity, and anti-swelling behavior. Excessive HCl led to a stiffening effect	Cut as needed	Ren et al. (2022)
Skin sensor	0–5 N	10 mV/N	Four flex sensors are embedded in molded silicone	Can effectively detect and estimate forces from multiple directions. The sensor may not work properly when the object is too thin or too sharp	Reference circle (diameter of 20 mm)	Subad et al. (2022)
Skin sensor	/	/	Discriminate both the direction and the magnitude of whisker deflection	Design and fabrication method is very simple, low-cost, quick. Stress whitening phenomenon may occur	Filament (initial length of 160 mm, diameter of 8 mm)	Gul et al. (2018)

## 6 Conclusion

Soft robot structures, combined with existing new materials and new control methods (Youssef et al., 2022), compared with traditional rigid robots in an underwater environment, as well as their lightness, dexterity, and various realization methods, have garnered considerable interest among scholars (Fang et al., 2022).

In the aspect of adhesion, soft tissue can be active or passive, and the adhesion is firm. Its excellent adhesion structure and underwater adaptability can be well bonded to the adsorbed surface (Chen et al., 2020). However, some problems remain to be solved in the aspect of the sucker. First, the adhesion performance of an object with an extremely rough surface is not good. Second, the surface with stains such as oil will have a negative impact on the adhesion capacity. Finally, the sucker will have a certain fatigue after repeated deformation (Kang et al., 2021).

In terms of the gripper, the soft grasping mechanism has a good grasping performance for vulnerable samples, and its diversified degrees of freedom are also very attractive (Rus and Tolley, 2015). However, the materials for soft grippers need to be further developed, and the structure and processing technology should be simplified. The control strategy also needs to be further studied and explored in combination with the material and motion characteristics of the specific mechanism (Wang and Cui, 2021).

In terms of the actuator, the underwater driving prototype has the advantages of low noise, making it more suitable for the habitat environment of underwater organisms and incorporating multi-functional composite mechanisms (e.g., transparency and low power advantages), which has attracted several scholars to study (Fu et al., 2021). However, further research is needed on the construction materials and testing of some prototypes in deep water (below 2,000 m), and the driving strategy can be further optimized and developed (Sun et al., 2022).

In terms of the soft sensor, as the most widely used for new materials, the fish-like lateral line and skin-like sensing devices can be used in several underwater applications. However, the properties of the new materials should be studied, including mechanical and chemical properties. In addition, the signal should be collected and quantified. Finally, a self-feedback correction is performed to be formally put into application (Won et al., 2021).

In this paper, several aspects of the development of the underwater soft robot are briefly introduced from the perspective of application. By starting from the common marine organisms, several main motion modes of the underwater soft robot are analyzed (viz., adhesion, grasping, driving, and sensing). The most common adhesion is the morphological biomimetics of clinging animals, such as octopus, remora, and clingfish. In addition, the gripper mainly studies and analyzes the behavior of predation and capture of organisms. In the aspect of actuators, it is briefly introduced that the tail drive of the swinging fish, the jet drive of the jellyfish, and the crawling drive of the starfish spider. Finally, some applications of biomimetic underwater sensors in the field of sensing are briefly introduced. This paper aims to help readers quickly understand the recent progress in different aspects of underwater soft mechanisms in order to guide engineers and technicians to a comprehensive understanding of specific problems in specific fields.
